# 儿童慢性髓性白血病酪氨酸激酶抑制剂停药的研究进展

**DOI:** 10.3760/cma.j.issn.0253-2727.2023.02.019

**Published:** 2023-02

**Authors:** 方圆 郑, 淼 王, 乐萍 张, 倩 江

**Affiliations:** 1 北京大学人民医院儿科，北京 100044 Department of Pediatrics, Peking University People's Hospital, Beijing 100044, China; 2 北京大学人民医院血液科、北京大学血液病研究所，北京 100044 Department of Hematology, Peking University People's Hospital, Peking University Institute of Hematology, Beijing 100044, China

儿童慢性髓性白血病（CML）是一种罕见的骨髓增殖性肿瘤，年发病率为（0.6～1.2）/100万，特征为9号与22号染色体易位产生Ph染色体，即t（9;22）（q34;q11），该染色体在分子水平上形成BCR-ABL融合基因。酪氨酸激酶抑制剂（TKI）能有效地抑制BCR-ABL激酶底物中酪氨酸残基的磷酸化，使该酶失活，通过阻止一系列的信号传导进而引起BCR-ABL阳性的细胞凋亡。其问世彻底改变了CML的治疗模式，也极大改善了CML患者的预后，CML患儿一线伊马替尼治疗的5年总生存率可达97％，预期寿命与正常人群相近[1]–[3]。虽然大多数CML患儿对TKI的治疗耐受性良好，但长期甚至终生服用仍可影响患儿的生活质量[4]，另外，由于儿童生长发育的特殊性，在关键阶段用药可能会影响到骨骼生长、生育能力等。鉴于近年成人CML的治疗目标已经从延缓疾病进展、改善生存质量扩大为追求无治疗缓解（treatment free remission，TFR），且有临床研究证实部分CML成人患者能够成功维持TFR状态，因此，尝试TKI停药对CML患儿来说具有非常大的吸引力。但与成人CML相比，儿童CML疾病侵袭性更强，该临床特点使得CML患儿的TKI停药成功率较成人更低，不建议CML患儿盲目停药。目前儿童TKI停药临床研究数据较少，我们结合成人TKI停药的研究结果对CML患儿的TKI停药进行综述。

1. CML患儿TKI停药的可能性：目前成人停药数据显示，伊马替尼或二代TKI治疗获得深度分子学反应［deep molecular response，DMR，其中包括MR4.0（BCR-ABL^IS^≤0.01％）、MR4.5（BCR-ABL^IS^≤0.0032％）、MR5.0（BCR-ABL^IS^≤ 0.001％）］2年以上患者停止TKI治疗维持分子学反应比例为40％～60％[5]–[7]。

关于CML儿童TKI停药后长期分子学缓解的研究，仅有少量的临床数据。日本儿童白血病/淋巴瘤研究小组（Japan Pediatric Leukemia/Lymphoma Study Group，JPLSG）[8]对22例诊断年龄<14岁、初诊时为慢性期（CP）、接受伊马替尼或二代TKI治疗、TKI治疗持续≥42个月、MR4.0持续≥25个月的CML患儿进行了停药研究，停药12个月时TFR成功率50％。一项多中心的“STOP IMAPED”研究[9]对14例应用伊马替尼治疗、伊马替尼治疗持续>45个月、DMR持续>19个月的CML患儿进行停药观察，有4例患儿在停药中位时间28（24～66）个月时仍处于DMR。儿童CML国际注册中心[10]对18例起病为CML-CP、应用伊马替尼>32个月、MR4.0持续≥2年的CML儿童进行停药研究，在停药后6、12、36个月TFR成功率分别为61％、56％和56％。法国和德国儿童CML登记处[11]回顾性分析6例初诊时CML-CP、Sokal评分低危、应用伊马替尼治疗16～73个月的CML患儿的停药数据，停药后的2～6个月内，6例患儿的BCR-ABL转录水平明显升高，5例丧失主要分子学反应（MMR，即BCR-ABL1^IS^ ≤0.1％），另1例失去DMR保留MMR。Moser等[12]报道2例起病CML-CP、接受伊马替尼治疗、分别MR4.0持续>31和27个月的CML患儿成功停药，患儿在停药48个月和19个月时仍持续为MR4.0。

意大利研究[13]–[14]为减少CML儿童的TKI药物不良反应、提高治疗依从性，对15例7～17岁CML患儿进行间歇性伊马替尼治疗研究（ON/OFF计划），具体为口服伊马替尼340 mg·m^−2^·d^−1^，每个月给药3周，休息1周，3例在间断伊马替尼治疗>16个月后予以停药，随访时仍为持续分子学反应。Giona等[13]对6例CML-CP患儿进行伊马替尼间歇治疗，其中有3例在DMR持续>72个月时停用伊马替尼治疗，随访34、34和54个月时均维持分子学反应。Jeyaraman等[15]报道1例伊马替尼治疗、DMR持续>4年的13岁CML男孩停药成功，该患儿在治疗期间出现身体质量指数（BMI）逐渐达到了36.5 kg/m^2^，后在医师指导下逐渐减停伊马替尼，起初为隔天用药一次，持续2个月，后为每周用药两次，持续1个月，之后停药，停药后20个月仍为持续DMR，且BMI降至28.5 kg/m^2^。

从以上的研究可以看出，目前儿童CML停止TKI治疗的临床试验病例数少，且部分为个例报道，提示虽然儿童CML存在TKI停药后持续分子学反应的可能性，但总体上成功率较低，因此，儿童CML停药需谨慎，不应轻易尝试停止TKI治疗。

2. TKI停药患者筛选：在成人CML中，不同专家组的TFR指南稍有差异。美国国家综合癌症网络（NCCN）2020[16]、欧洲白血病网（ELN）2020[17]、M.D.安德森癌症中心（MDACC）[18]CML指南建议临床试验外，满足“>18岁、起病为CML-CP、为BCR-ABL（P210）转录本、既往无TKI耐药史、能够规范进行分子学检测（采用国际标准化定量）、停药失败后能够获得及时再治疗以及准确的再治疗后分子学检测”者，需在有经验的临床医师的指导下才能进行TFR尝试。目前停药试验显示获得持续MR4/MR4.5以上分子学反应，并且持续>2年是目前停药试验的前提条件，仅获得完全细胞遗传学反应或者MMR的患者停药后均迅速分子学复发。然而，这些标准只为成人CML建立，它们是否能用于CML患儿还有待证实。在上述CML患儿停药试验中，CML起病时均为CML-CP，多为e13a2、e14a2转录本，无TKI耐药史，且获得DMR持续时间最低19个月、接受TKI药物治疗持续时间最低40个月才进行停药，在经过严格筛查停药目标人群的情况下，仍有超过一半的儿童在停用伊马替尼后经历了分子学复发，因此，CML患儿的停药治疗需要谨慎进行。若在进行TKI停药临床试验中，停药条件可以参考更为严格的MDACC停药标准[18]：BCR-ABL转录本为e13a2或e14a2、初诊时处于CP、一线TKI治疗达最佳反应、持续TKI使用时间>6～8年、DMR持续时间>3年，并且能够在停药的前12个月内每6～8周、第2年内每2～3个月、2年之后每4～6个月进行分子学检测。

3. 成功实现TFR的影响因素：明确TKI停药的预测因素是日后指导停药治疗的关键，在成人停药试验中发现停药前分子学反应的深度和持续时间、Sokal评分是影响TFR成功的主要因素[19]，并且认为男性、高龄、TKI治疗的快速反应和e14a2转录本类型的表达也是停药后复发的低风险因素[20]。在儿童停药研究中，JPLSG研究[8]显示TFR成功与停伊马替尼前血药浓度相关，伊马替尼血浆浓度越高，复发风险越高。Millot等[10]研究认为年龄、性别、Sokal和ELTS评分、伊马替尼治疗时间和停药前DMR时间对TFR无影响。此外，在成人停药研究中发现采取阶段性减少TKI用量会提高TFR成功率。Giona等[13]、Jeyaraman等[15]报道的患儿，停药措施也为逐渐减停伊马替尼，在停药20个月仍为持续DMR，但逐渐减停的停药方案是否为停药成功的影响因素，还需大样本进一步观察。

4. 停药后相关反应：在成人TKI停药试验中，已有报道以不同程度的肌肉骨骼疼痛和瘙痒为特征的“伊马替尼停药综合征”。ENESTTop研究[21]在TFR期间观察到42％的成人患者出现肌肉疼痛、四肢疼痛、关节痛、骨痛、脊柱痛等不良事件。在CML患儿中，JPLSG研究[8]的22例患者在停用TKI后，3例出现1级肌肉骨骼或关节疼痛，6例出现1～3级磷酸肌酸激酶升高。STOP IMAPED研究[9]的14例患者在停药后未发生任何安全问题。

5. 分子学复发及重启TKI治疗情况：在成人CML停药试验中多将MMR丧失作为分子学复发。CML患者停药后复发主要发生于早期即6个月内，但也有15％的患者在停药后6年出现晚期复发[22]。在CML患儿停药研究中，JPLSG研究[8]将分子学复发定义为至少丧失MMR，22例CML患儿中有11例出现分子学复发，复发均发生于停药后3个月内，重启TKI治疗后中位64（25～196）d全部再次达到MR4.0。STOP IMAPED研究[9]将分子学复发定义为丧失MR4.0，该研究14例CML患儿中有10例在停药后6个月内出现分子学复发，分别重启伊马替尼或达沙替尼治疗，所有复发患儿在中位8（1～34）个月内重新获得DMR。儿童CML国际注册中心研究[10]中，7例停药后分子学复发的CML儿童，有6例在重启伊马替尼治疗的2.5～18个月获得MR4.0，1例获得MMR。由此可见，虽然儿童CML停药后复发重启TKI治疗后也可再次获得分子学缓解，但由以上研究可以看出，即使是在严格条件限制下的TKI停药，分子学复发的比例仍较高。因此对于儿童CML的TKI停药一定要谨慎对待，对于已经符合停药条件并尝试停药者，一定要密切监测分子学水平，在出现分子学复发后，立即重启TKI治疗。

6. 总结：TFR已经成为儿童CML治疗研究的新目标，但目前儿童的TKI停药成功率低，只有小部分患儿获得持续的TFR，因此，在儿童CML的诊疗中，不能盲目地将TKI停药作为治疗目标，在没有更多循证医学证据的情况下，TKI停药不宜在儿童CML中推广，即使进行TKI停药临床试验，也需要谨慎确定停药目标人群、严格把握停药时机、及时准确地监测停药分子学反应。

## References

[1] Smith SM, Hijiya N, Sakamoto KM (2021). Chronic myelogenous leukemia in childhood[J]. Curr Oncol Rep.

[2] Suttorp M, Millot F, Sembill S (2021). Definition, epidemiology, pathophysiology, and essential criteria for diagnosis of pediatric chronic myeloid leukemia[J]. Cancers (Basel).

[3] Millot F, Guilhot J, Suttorp M (2017). Prognostic discrimination based on the EUTOS long-term survival score within the International Registry for Chronic Myeloid Leukemia in children and adolescents[J]. Haematologica.

[4] Zheng F, Dou X, Zhang L (2022). Health-related quality of life in children with chronic myeloid leukemia in the chronic phase[J]. J Cancer Res Clin Oncol.

[5] Mahon FX, Réa D, Guilhot J (2010). Discontinuation of imatinib in patients with chronic myeloid leukaemia who have maintained complete molecular remission for at least 2 years: the prospective, multicentre Stop Imatinib (STIM) trial[J]. Lancet Oncol.

[6] 朱 晓健, 游 泳, 段 明辉 (2018). 慢性髓性白血病患者酪氨酸激酶抑制剂自动停药的多中心回顾性研究[J]. 中华血液学杂志.

[7] Etienne G, Guilhot J, Rea D (2017). Long-term follow-up of the french stop imatinib (STIM1) study in patients sith chronic myeloid leukemia[J]. J Clin Oncol.

[8] Shima H, MD PhD, Kada A (2019). Discontinuation of tyrosine kinase inhibitor in children with chronic myeloid leukemia (JPLSG STKI-14 study)[J]. Blood (Annual ASH meeting abstract).

[9] de Bruijn CMA, Millot F, Suttorp M (2019). Discontinuation of imatinib in children with chronic myeloid leukaemia in sustained deep molecular remission: results of the STOP IMAPED study[J]. Br J Haematol.

[10] Millot F, Suttorp M, Ragot S (2021). Discontinuation of imatinib in children with chronic myeloid leukemia: a study from the international registry of childhood CML[J]. Cancers(Basel).

[11] Millot F, Claviez A, Leverger G (2014). Imatinib cessation in children and adolescents with chronic myeloid leukemia in chronic phase[J]. Pediatr Blood Cancer.

[12] Moser O, Krumbholz M, Thiede C (2014). Sustained complete molecular remission after imatinib discontinuation in children with chronic myeloid leukemia[J]. Pediatr Blood Cancer.

[13] Giona F, Saglio G, Moleti ML (2015). Treatment-free remission after imatinib discontinuation is possible in paediatric patients with chronic myeloid leukaemia[J]. Br J Haematol.

[14] Giona F, Malaspina F, Putti MC (2020). Results and outcome of intermittent imatinib (ON/OFF schedule) in children and adolescents with chronic myeloid leukaemia[J]. Br J Haematol.

[15] Jeyaraman P, Naithani R (2020). Discontinuation of imatinib in a child with chronic myeloid leukemia[J]. J Pediatr Hematol Oncol.

[16] Deininger MW, Shah NP, Altman JK (2020). Chronic myeloid leukemia, version 2.2021, NCCN clinical practice guidelines in oncology[J]. J Natl Compr Canc Netw.

[17] Hochhaus A, Baccarani M, Silver RT (2020). European LeukemiaNet 2020 recommendations for treating chronic myeloid leukemia[J]. Leukemia.

[18] Jabbour E, Kantarjian H (2022). Chronic myeloid leukemia: 2022 update on diagnosis, therapy, and monitoring[J]. Am J Hematol.

[19] Dulucq S, Astrugue C, Etienne G (2020). Risk of molecular recurrence after tyrosine kinase inhibitor discontinuation in chronic myeloid leukaemia patients: a systematic review of literature with a meta-analysis of studies over the last ten years[J]. Br J Haematol.

[20] Saifullah HH, Lucas CM (2021). Treatment-free remission in chronic myeloid leukemia: can we identify prognostic factors?[J]. Cancers (Basel).

[21] Mahon FX, Boquimpani C, Kim DW (2018). Treatment-free remission after second-line nilotinib treatment in patients with chronic myeloid leukemia in chronic phase: results from a single-group, phase 2, open-label study[J]. Ann Intern Med.

[22] Ross DM, Branford S, Seymour JF (2013). Safety and efficacy of imatinib cessation for CML patients with stable undetectable minimal residual disease: results from the TWISTER study[J]. Blood.

